# Experiences of patients with chronic diseases during the COVID-19 pandemic in the North West province, South Africa

**DOI:** 10.4102/safp.v65i1.5643

**Published:** 2023-06-29

**Authors:** Sheillah H. Mboweni, Patrone R. Risenga

**Affiliations:** 1 Department of Health Studies, College of Human Sciences, University of South Africa, Pretoria, South Africa

**Keywords:** patients with chronic diseases, COVID-19, experiences, exploring, chronic disease, healthcare facilities

## Abstract

**Background:**

Patients with chronic diseases (PWCDs) were severely affected by the coronavirus disease 2019 (COVID-19) pandemic, as they were prevented from making the necessary visits to health facilities for medical review and to collect their medication. The emergence of the health crisis and inadequate access to quality care affected chronic care management. The perspectives of PWCDs are not known, and therefore the research on which this paper is based sought to investigate the lived experiences of these patients during the COVID-19 pandemic.

**Methods:**

A qualitative phenomenological design was used to obtain the lived experiences of PWCDs identified for participation in the study by means of purposive sampling. Patients’ experiences were obtained during individual structured interviews, and a checklist was used to gather patient characteristics from their files.

**Results:**

Three themes emerged from the study findings, namely poor healthcare services, the socio-economic impact of the COVID-19 pandemic, and the psychological impact of the COVID-19 pandemic. The COVID-19 pandemic had devastating effects on PWCDs, in that they experienced barriers to accessing quality chronic care services and suffered psychological and financial difficulties that affected their health, life, needs and expectations.

**Conclusion:**

Policymakers should consider PWCDs when responding to a public health concern in the future.

**Contribution:**

The study findings may have an impact on future policies regulating the management of chronic diseases during epidemics, in order to improve patient health outcomes and satisfaction with healthcare services and the chronic care model based on the experiences of PWCDs.

## Background

Chronic diseases are the leading causes of death and disability worldwide and account for 80% of premature deaths among people between the ages of 30 and 69 years. The threat is growing annually globally, especially in low- and middle-income countries (LMICs), and requires a new approach by national leaders and the public health community.^1^ Investment in the prevention, management and control of chronic diseases should aim at reducing premature deaths by one-third world-wide through comprehensive and integrated action at the country-led level and part of the 2030 agenda for Sustainable Development Goals.^2^ The emergence of severe acute respiratory syndrome coronavirus 2 (SARS-CoV-2), known as coronavirus disease 2019 (COVID-19), placed additional pressure on a healthcare system already overwhelmed by the burden of disease. Globally, human immunodeficiency virus (HIV) and acquired immunodeficiency syndrome (AIDS), tuberculosis (TB), hypertension (HPT) and diabetes mellitus (DM) are the diseases and conditions placing particular strain on healthcare systems.^3^ According to the World Health Organization (WHO),^4^ in 2013, 38 million out of 56 million deaths globally were reported to be associated with non-communicable diseases (NCDs); this figure has risen from 59% in 2002 to 69% in 2010. On the other hand, the number of people living with HIV (PLHIV) increased from 31 million in 2010 to 38 million in 2018, adding to the burden of chronic diseases.

The COVID-19 pandemic was highly politicised, and during that time chronic disease management services were suspended in many countries, including South Africa.^5,6^ This approach is contrary to the WHO approach of investing in the prevention, control and management of chronic diseases and to the White Paper for the transformation of the healthcare system in South Africa including human rights in the African region. According to the WHO,^7^ NCDs are usually neglected during emergencies, with the focus being purely on infectious disease outbreaks, and NCDs are not being considered part of the emergency response. Chronic diseases are described by the WHO^7,8^ as diseases of long duration and slow progression; these include NCDs and communicable diseases (CDs) such as HIV, as the latter has changed from being a devastating epidemic to a manageable chronic disease through the life-long application of antiretroviral therapy (ART). A sudden shift in international, regional and national health priorities was indeed observed when countries were endeavouring to curb the spread of COVID-19.^9^

All stakeholders, including emergency planners, emergency care professionals, and policymakers tasked with emergency response and preparedness must be instructed not to overlook patients with chronic diseases (PWCDs) during planning. In response to the pandemic, most countries implemented stringent lockdown regulations, which resulted in limited access to or utilisation of healthcare services. Human immunodeficiency virus and NCDs are more prevalent in LMICs and mainly in sub-Saharan Africa.^10^ However, COVID-19 mainly affected developed countries. As of July 2022, South Africa had reported more than 3 995 400 cases and more than 105 000 deaths.^11,12^ The White Paper for the Transformation of the Health System in South Africa focuses on the need for accessible, comprehensive and integrated healthcare services; however, with healthcare services of this nature having been disrupted by the COVID-19 outbreak, the researchers identified the need for a study to investigate the experiences of PWCDs in a high-density mining and farming district in South Africa during the COVID-19 pandemic.

Chronic diseases contribute to the burden of diseases in both the public and private healthcare sectors. South Africa was one of the countries to implement stringent lockdown regulations early on, which seriously affected the delivery of healthcare services. Travelling and movement were restricted, which had repercussions for the operation of transport services and many other businesses.^11,13^ The risk of complications related to COVID-19 was reported to be high among PWCDs because of comorbidity, and this instilled fear among this group of patients. The sudden evolution of the pandemic disrupted healthcare services for people with chronic diseases, negatively affecting the achievement of their treatment goals and objectives. According to Organization for Economic Co-operation and Development (OECD)^12^ uncertainty regarding the implications of COVID-19 and the stringent lockdown regulations affected patients’ access to care, management, treatment and support. Restrictions on movement were enforced by police, and there were frequent and abrupt closures of health facilities because of the volume of COVID-19 cases requiring treatment. The responses of PWCDs to the COVID-19 pandemic, including the alterations to the management of their chronic illnesses and their expectations, are not known. Therefore, the researchers felt that it would be important to conduct a study to explore and describe the experiences of PWCDs during the COVID-19 pandemic in South Africa to make recommendations to policymakers for enhanced quality patient-centred care should a similar situation arise in the future.

## Methods

A phenomenological qualitative study was conducted to investigate the lived experiences of PWCDs during the lockdown imposed in response to the COVID-19 pandemic. The phenomenological design is the best approach to capture the lived in-depth narrative experiences from the perspective of participants to construct understanding and meaning while setting aside the researcher’s own beliefs.^14,15,16^ The study population comprised all PWCDs in the selected public healthcare (PHC) facilities of the district. A non-probability purposive sampling method was used to hand-pick 28 PWCDs who were willing to share their first-hand experiences from the selected high-volume public health facilities, situated in a high-density district comprising informal settlements and farming and mining activities in the North West province. The district is predominantly rural, characterised by a large number of migrant workers. The first participants were purposively selected, followed by a consecutive sampling of all participants attending the chronic diseases clinic and meeting the inclusion criteria.^14,15,17,18^ All chronic disease patients who were 18 years and above and mentally stable according to the inclusion criteria were included in the study. Patients who came for clinical review at the chronic diseases clinic, but whose vital signs were higher than expected levels, who were very ill and necessitated doctor’s consultation and referral to the hospital were excluded from the study to prevent delays to care.

## Data collection

Data were collected through an unstructured individual face-to-face interview. Written informed consent was obtained from participants who were able to read and write, and verbal informed consent from those unable to read and write. Interviews were conducted with each participant in a private room to capture their experiences; this setting was selected to afford each participant privacy in which to express their views, feelings and experiences. The interviews were recorded on audio tape. A checklist was used to capture information reviewed from the participant’s records or files; this included vital signs related to chronic diseases, whether the patient’s condition was either stable or unstable, compliance with the appointment system and demographic data. According to the American hospital association,^19^ a stable condition means the patient is in good condition, and vital signs like heart rate, blood pressure and body temperature are steady and within normal, conscious and comfortable and unlikely to deteriorate soon; therefore, they no longer need frequent clinical management, while unstable patients refer to patients with unsteady vital signs and not within normal limits and are very ill. The development of the checklist was guided by Andersen’s behavioural model of health services use in chronic disease services and support.^20^ Field notes were used to capture non-verbal responses, including the emotions displayed by the participants. The data collection process was carried out from April to June 2022. Data saturation was reached with 28 patients. According to Brink et al.^15^ and Creswell and Creswell^18^ data saturation occurs when additional participants provide no new information and when themes that emerge become repetitive; this can be reached with 12–25 participants.^1,16^ The field notes and audio recordings were kept under lock and key, apart from the consent form, and participant numbers were used instead of names.

## Data collection procedure

During data collection, the COVID-19 pandemic facility infection prevention and control (IPC) guidelines and policies were applied by ensuring that the room was well ventilated, that a distance of 1.5 m between people was maintained and that hand sanitiser and masks were available. The facility’s operational managers and nurses managing chronic disease were used as gatekeepers to access patients from the facility by e-mailing consent forms and information leaflets to patients, explaining in detail the purpose, benefits and risks of the study. The following question was posed to participants by the researcher, who collected data: What is your lived experience of the COVID-19 pandemic regarding the management of your chronic diseases? This was followed by further interaction to assist participants in obtaining clarity if they required this. The patients were interviewed in their language, Setswana, and their responses were translated into English during transcription. The researcher can understand and speak Setswana, having worked in the province for 8 years. The duration of each interview was between 30 min and 45 min.^21^

## Data analysis

Data analysis was done concurrently with data collection. Thematic analysis was carried out to analyse the lived experiences of PWCDs using the four steps of descriptive phenomenology, namely bracketing, intuiting, analysing, and describing.^11^ In the *bracketing phase*, the researchers strove to put aside their beliefs and opinions about the management of chronic disease patients during the COVID-19 pandemic to confront data by maintaining a reflexive journal covering possible role conflict and making notes of interest and the use of gatekeepers to ensure neutrality. Data were transcribed verbatim. The researchers read and re-read the transcribed data until they reached immersion and began coding similar data into categories and themes. In the *intuiting phase*, the researchers remained open to the meaning by sharing the transcribed data and emerging themes with an experienced researcher to ensure quality, and in the *analysis phase,* the researchers extracted significant statements, began categorising data, made sense of the essential meaning and reached a consensus of the themes and sub-themes. Finally, in the *descriptive phase*, the researchers came to understand and defined the phenomenon under study.^11^

## Trustworthiness in qualitative research

Trustworthiness is a way of ensuring data quality or rigour in qualitative research.^14^ The trustworthiness of the study overall was guaranteed through the use of the model of Lincoln and Guba (1985), as cited in,^14,15,17,18^ consisting of the criteria to ensure the credibility, dependability, confirmability and transferability of the study. Various strategies were used to enhance trustworthiness, including the use of the phenomenological design, purposive sampling methods, audio recording of the interviews to obtain participants’ lived experiences and reviewing of the participants’ files to obtain a clearer picture and meaning of the phenomenon under study. Reflexivity as a means to enhance credibility refers to the researcher’s self-awareness and was applied as a means to guard against personal bias and to enhance the quality of the study.^11,13,14^ Dependability was enhanced by implementing strategies to enhance credibility, including spending a reasonable length of time with each participant (30–45 min) and conducting a member check, by which the researcher went back to participants to verify whether their experiences had been captured correctly. Transferability was enhanced by collecting in-depth lived experiences of the PWCDs from different facilities in the district until data saturation was reached. A pilot study was conducted in a different facility from the one in which the study was conducted. Conformability was enhanced by keeping audit trails of the audio-recorded interviews and careful documentation of field notes and file review information using a checklist and making these available to an experienced researcher to ensure quality.^17,18^

### Ethical considerations

The study was approved by the Unisa College of Human Sciences Ethics Committee and assigned reference number 90476050-CREC_CHS_2021. Permission to conduct the study was obtained from the North West Provincial Department of Health and Bojanala district institution review board. Both verbal and written voluntary informed consent was obtained from the participants. Numbers were used instead of patients’ names. Furthermore, in terms of the *Protection of Personal Information (POPI) Act*, which came into effect in April 2020 in South Africa, and to comply with ethical principles, no information that would make it possible to identify the participants was used. The recordings on audio tape were kept under lock and key separately from the consent forms, and field notes were saved electronically and password protected. To ensure anonymity, confidentiality and data safety, this data can only be accessed by the researcher. The participants were informed of their right to withdraw at any stage of the study without penalty. The interviews were conducted in a private room where the participants were not disturbed and where they could not be seen or overheard.

## Results

The study revealed three themes, namely poor healthcare services, the socio-economic impact of the COVID-19 pandemic on PWCDs and the psychological impact of the pandemic on PWCDs, as indicated in Table 1. The demographics of the participants and the selected public health facilities were also collected from patient files using the checklist, supplemented by the interviews and are summarised in Table 2.

**TABLE 1:** Themes and subthemes that emerged from the study findings.

Theme	Subthemes	Sub-subtheme
1. Poor healthcare services	1.1. Barriers to accessing healthcare services	Long waiting hours
		A limited number of patients seen per day
		Health facility closure
		Staff shortage and staff attitude
		Lack of knowledge about COVID-19
		Shortage of medicine and supplies
	1.2. Poor chronic care management	Substandard chronic care management
	1.3. Lack of psychosocial support	Lack of education, counselling, and support
2. Psychological impact of COVID-19 on PWCDs	2.1. Mental health problems experienced by PWCDs	Fear
		Stress
		Depression
3. Socio-economic impact of COVID-19 on PWCDs	3.1. Job loss	
	3.2. Failure to provide for basic needs	
	3.3. Migration to other provinces and countries	

COVID-19, coronavirus disease 2019; PWCDs, patients with chronic diseases.

**TABLE 2:** Demographics of the participants and facility.

Demographics	Frequency (*n*)	%
**Gender**		
Female	21	75.00
Male	7	25.00
**Age in years**		
18–22 years	2	7.14
23–27 years	2	7.14
28–32 years	0	0.00
33–37 years	3	10.71
38–42 years	6	21.43
43–47 years	8	28.57
48–52 years	1	3.57
53–57 years	2	7.14
58–62 years	2	7.14
62 and above	2	7.14
**Residential area**		
Rural	24	85.71
Urban	4	14.28
HPT	7	25.00
**Type of chronic disease**		
DM	3	10.71
Asthma	0	0.00
HIV on ART	13	46.40
Cardiac condition	1	3.60
With comorbidity	4	14.30
Employed	5	17.85
**Employment**		
Self-employed(selling)	2	7.14
Unemployed	21	60.71
Employment	5	17.90
**Source of income**		
Government grant	4	14.28
Selling	2	7.14
**Proximity to the health facility (10 kms)**		
+10 km	10	35.50
Less than 10 km	18	64.20
**Facility operating hours**		
24 hours	20	71.42
8 hours	0	0.00
12 hours	8	28.57
**Appointment system**		
No	4	14.28
Yes	24	85.70
**Support system**		
Buddy/friend	3	10.71
Relative (partner/family)	24	85.71
CBO	1	3.60
**Medical aid or health insurance**		
Yes	1	3.57
No	27	96.40
**Average waiting hour**		
3 hour	0	0.00
4 hour or more	28	100.00
Stable	18	64.30
**Patient outcomes**		
Not stable	10	35.70
Space fast lane (SFL)	1	3.57
**Decanted**		
External pick-up point	3	10.71
Adherence club (AC)	0	0.00

**Total**	**28**	**100.00**

HPT, hypertension; DM, diabetes mellitus; HIV, human immunodeficiency virus; ART, antiretroviral therapy; CBO, community-based organisation.

## Participant demographics

Table 2 shows the demographics of both the facility and the participants who took part in the study. A total of 28 patients from the four high-volume facilities were interviewed, and data saturation was reached with this sample. The majority of the participants (75%) were female, and the remaining 25% were male. In terms of age, 32.1% were between the ages of 43 and 47 years, with the lowest percentage (3.5%) being between the ages of 48 and 52 years. Most participants were PLHIV on ART (46.4%), the next largest group comprising those with HPT (25%); the smallest number were participants with cardiac conditions and the lowest number of participants with comorbidity were at least 14.3%. The majority of the participants (85.7%) were from rural areas, and only 14.3% were from urban areas. Most participants (75%) were unemployed; 17.5% worked for an employer and 7.1% were self-employed. Sources of income were government grants (14.3%), employer (17.9%) and selling (7.1%).

In terms of distance from a health facility, 35.7% stayed farther than 10 km away and 64.3% stayed closer than 10 km to the facility. With regard to operating hours, 71.4% of the health facilities operated 24 h a day, while the remaining 24.6% operated 12 h a day. All the participants (100%) reported spending more than 4 h at the health facility to receive service. In terms of appointments, 85.7% of the participants visited the facility on the return date written on the card, while 14.3% came to the facility at a time other than their scheduled appointment. Most patients (85.7%) received the support of their relatives, which included partners, children, and parents, while 10.7% received the support of buddies or friends, and 3.6% received the support of community-based organisations (CBOs). Most participants (96.4%) had no medical aid or health insurance, and only 3.6% did in fact have health cover. Based on vital signs, the conditions and clinical outcomes of 64.3% of the participants were stable, while those of 35.7% of the participants were not stable, and some were referred to be seen by a medical doctor. A large number of participants (85.7%) were not decanted (Decanting refers to the process of getting stable patients who do not need to be seen by a healthcare provider more than once a year to collect their treatment from a location inside or outside a clinic to which the health department will courier it through the central chronic medicines dispensing and distribution programme [CCMDD]) with a few of the decanted participants (3.6%) being in the space fast lane (SFL) (SFL is a facility pick-up point where all decanted patients are fast-tracked for the collection of their medication in order to reduce facility congestion and long waiting times) and 10.7% external pick-up point. (External pickup [Ex-PUP] points are areas closer to PWCDs’ homes or workplaces that provide a more convenient option for medication collection outside the healthcare facilities, dispensed and distributed via the CCMDD programme, and such points should be contracted by the department of health). Adherence clubs (Adherence clubs [ACs] are a group-level intervention of decanted PWCDs in the same cohort who can support and learn from each other before collecting their medication in order to improve retention of care and sustain clinical outcomes) were disbanded in compliance with the COVID-19 pandemic regulations. Space fast lane is a facility pick-up point where all decanted patients are prioritised for medication collection in order to reduce facility congestion and long wait times.

Participants revealed that they faced barriers such as inadequate access to healthcare services because of frequent and abrupt closure of health facilities, long waiting hours, a limited number of patients seen per day, substandard chronic care and management by healthcare providers, shortage and attitude of staff, a lack of knowledge and shortage of medicines and supplies.

### Theme 1: Poor healthcare services

Participants expressed disappointment in the poor healthcare services they received during the COVID-19 pandemic, especially during the level 5 and 4 lockdowns, which created barriers to accessing health services; these included long waiting hours, a limited number of patients seen per day, closure of health facilities, shortage and attitude of staff, a lack of knowledge related to COVID-19 and shortage of medicine and supplies.

#### Subtheme 1.1: Barriers to accessing healthcare services

**Long waiting hours:** Participants revealed that they had to wait for long periods to access chronic healthcare services and medication, which led to missed appointments. A participant stated:

‘I have to come early in the morning at 06h00 to queue for the file and I just received the service at 11h15, it is not good at all’. (P2, female, 25 March 2022)

**A limited number of patients seen per day:** Participants reported that as the result of a shortage of staff, medical staff were able to see only a limited number of people per day; this deprived patients of access to chronic care and also contributed to missed appointments. In the words of one of the participants:

‘I had to queue two days in succession because I was cut off the line, staff indicating that some nurses are off sick of contracting the COVID-19 infection’. (P3, male, 25 April 2022)

**Health facility closure:** Participants expressed concern about not receiving treatment for their chronic condition or not being attended to when they came for review because the facility was closed and they had not been informed of this:

‘I came early only to find that the facility is closed, very frustrating’. (P22, male, 17 May 2022)

‘It was my return date for a check-up and found that the facility was not opened’. (P28, male, 23 June 2022)

**Staff shortage and staff attitude:** Participants reported staff shortages at the facilities and that they had to wait to be seen by two nurses, which contributed to long waiting hours. One participant was recorded as saying:

‘How can the government not employ more staff when they know others are sick’. (P6, male, 26 April 2022)

Apart from the shortage of staff, participants complained of staff attitude, from reception to nurses:

‘The staff was very rude, they do not treat us as human beings’. (P11, female, 17 May 2022)

Another participant had the following to say:

‘We need staff and nurses who can greet us with a smile and treat us with respect, we are also human beings’. (P2, female, 25 March 2022)

Another participant reported:

‘Nurses scolded us, asking why are we so many at the clinic, don’t we know there is COVID-19, we must stay at home’. (P18, female, 11 May 2022)

**Lack of knowledge about COVID-19:** Participants revealed that they lacked knowledge about COVID-19 and were given conflicting information, which left them confused and frustrated:

‘I came to the facility to ask about, what we heard from TV and radio and nurses were not having clear information indicating that they also do not have all the answers as it is a new illness’. (P20, female, 07 June 2022)

**Shortage of medicine and supplies:** Participants revealed that there was a shortage of medicine and supplies to manage their chronic conditions. One of the participants reported:

‘I felt like we are going to die, imagine given insulin without needles, where would I get it’. (P24, male, 23 June 2022)

Another participant stated:

‘I was told to buy my ART medication and was very expensive, could not afford it, was about R400’. (P13, female, 7 June 2022)

A participant explained:

‘My blood sugar level was not checked because there was no machine to check it’. (P7, male, 26 April 2022)

#### Subtheme 1.2: Poor chronic care management

**Substandard chronic care management:** Participants reported not receiving the chronic care they had received in the past. In the words of one of the participants:

‘Yhooo the quality was bad, BP and sugar were not tested, they just gave me medication’. (P4, male, 25 April 2022)

Another patient reported:

‘The nurse and the doctor did not touch me or do a physical examination; they were just at the distance’. (P8, female, 26 May 2022)

#### Subtheme 1.3: Lack of psychosocial support

**Lack of counselling, education and support:** The study revealed that education, counselling and support regarding chronic illness were lacking, and that participants felt neglected. The adherence clubs and CBOs providing counselling and support were disbanded in line with COVID-19 regulations to control the transmission of infection. A participant made the following observation:

‘Nurses and staff only talk about COVID-19 only, nothing about us’. (P9, male, 26 April 2022)

In the words of another participant:

‘I was diagnosed with HIV and was so shocked, stressed not knowing whom to talk to, they promise to talk to me over the phone’. (P26, female, 23 June 2022)

### Theme 2: Psychological impact of COVID-19 on patients with chronic diseases

Participants experienced mental health problems such as fear, stress and depression in relation to their conditions and COVID-19 complications.

#### Subtheme 2.1: Mental health problems experienced by patients with chronic diseases

**Fear:** Participants expressed their fear of contracting COVID-19 and complications related to co-morbidity, which resulted in their missing appointments:

‘My blood sugar was high, and refuse to be referred to the hospital because I thought COVID-19 is high in there as most die’. (P27, female, 23 June 2022)

Another patient was recorded as saying:

‘I was afraid to go to the clinic or travel in a taxi because of COVID-19 and missed my appointment’. (P21, female, 7 June 2022)

Another participant indicated that:

‘I was afraid about what we heard from the radio and TV [*television*] about death people of who are old or with co-morbidity’. (P15, female, 11 May 2022)

**Stress:** Participants experienced stress arising from their fear of contracting COVID-19, long waiting hours and shortages of medication and staff attitude, which led to depression in some of the patients:

‘My BP was high as I was stressing, I was thinking I will die when I was suffering from COVID-19 infections, as a chronic patient’. (P12, female, 7 May 2022)

As another participant explained:

‘COVID-19 brought me stress as I no longer sell fruits and vegetables to care for myself and family’. (P10, female, 26 May 2022)

**Depression:** The study revealed that PWCDs suffered from depression because the COVID-19 restrictions prevented visits to the elderly and people with chronic diseases because they were at risk of complications; they were therefore isolated and excluded from interaction with and participation in the community.

A participant shared the following experience:

‘I suffered depression because of the stress of being separated from my family as they were trying to protect me from getting COVID-19, it was tough’. (P13, female, 7 June 2022)

### Theme 3: Socio-economic impact of COVID-19 on patients with chronic diseases

Participants revealed that their socio-economic status was worsened by COVID-19 in that they lost their jobs and were unable to sell their goods and, in consequence, did not have money to buy food. Shops and nearby complexes were closed, and they were afraid to travel to buy food.

#### Subtheme 3.1: Job loss

The study revealed job losses among PWCDs arising from the fear of contracting COVID-19 and termination of contracts by employers; furthermore, people were not allowed to move around to sell goods or stock goods to sell.

As one of the participants explained:

‘I lost my piece job of washing clothes in my area’. (P5, female, 25 April 2022)

Another participant indicated:

‘I lost my contract from the mine because of COVID-19’. (P1, female, 25 April 2022)

#### Subtheme 3.2: Failure to provide for basic needs

The study revealed that participants failed to provide for their basic needs, such as a balanced diet, as they lost their jobs, small businesses were not active, most shops closed and movement and travelling were restricted. All of this negatively affected their nutritional status although good nutritional status forms part of the therapeutic management of chronic diseases. A participant reported:

‘I failed to buy groceries, vegetables, and fruits, and had to rely on my children and family’. (P3, male, 25 April 2022)

Another patient stated:

‘I failed to buy insulin needles as it was out of stock at the clinic’. (P6, female, 26 May 2022)

#### Subtheme 3.3: Migration to other provinces and countries

Some participants who were from neighbouring provinces and countries who were working and living in the district in order to engage in mining and farming activities decided to go back home, as they had lost their jobs and were afraid of contracting COVID-19. This also contributed to a large number of missed appointments. A participant explained:

‘I was no longer working and managed to go back home as I cannot afford to pay my rent and food’. (P14, female, 17 May 2022)

Another participant shared that:

‘I went back home to Mozambique because my husband lost his job’. (P17, female, 11 May 2022)

## Discussion

The study results are discussed under the following four headings: poor quality of chronic disease care and management, mental health problems, socio-economic impact, and difficulties in accessing healthcare services during the COVID-19 pandemic.

### Poor quality of chronic disease care and management

The participant demographics revealed participants who were relatively young, aged 37 years and below, to be suffering from chronic diseases that, if not treated properly, had the potential to lead to complications and premature death. This was supported by a study conducted by Syed et al.^22^ in Qatar, in which it was reported that the highest increases in the prevalence of NCDs were seen in a relatively young age group (30–49 years), although this increased with age and was higher in women, at 25%, than in men at 16.5% within this age group. The WHO^1^ report of 2021 indicates that more than 15 million persons between the ages of 30–69 years die from an NCD every year; 85% of these ‘premature’ deaths take place in low- and middle-income nations. Again, Belaunzaran-Zamudio et al.^23^ found that between 2000 and 2015, multi-morbidity increased from 30% to 40% and the annual prevalence of NCDs among people aged 50 years or older living with HIV rose from 32% to 68%.

The study also revealed HIV and/or AIDS, HPT, cardiac problems, and DM to be the leading chronic diseases. Comorbidity exposes patients with chronic disease to complications related to COVID-19 infections.^23^ According to the study findings, a greater percentage of women suffered from chronic diseases and were more likely to utilise healthcare services than men. According to OECD^12^ and Syed et al.,^22^ women are more likely than men to have multiple chronic diseases (41% vs 32% on average). Chronic disease prevalence rises with age, as expected. Furthermore, women endure the stress and burden of fulfilling their roles without the support and are vulnerable to gender-based violence and intimate partner violence, with one in three women globally experiencing violence.^24^

The study findings revealed problems relating to inadequate access to chronic care and other services and that the implementation of the appointment and decanting system is still lacking and should be enhanced to reduce long waiting hours and overcrowding in facilities. This was supported by Syed et al.^17^ and Liang and Zhao^24^ who state that the use of an appointment system increases patient satisfaction as it saves time, reduces congestion, guarantees time slots and eliminates excessively long waiting times. The study revealed that physical access to healthcare services remained difficult, as some patients had to travel more than 10 km to access treatment, and people in rural areas continue to suffer, as they do not have medical aid. A diagnostic report on access to quality healthcare since 1994 in South Africa indicated that the majority of people who use public health facilities live in rural areas, are not on a medical plan and belong to the lowest socio-economic classes.^25,26^

Long waiting times are also an obstacle despite the implementation of clinic initiatives such as an appointment system and can lead to a high level of missed appointments and patient service dissatisfaction. This was supported by a study conducted in South Africa that indicated that patients continue to wait longer than 4 h to receive services as a result of poorly administered policies and guidelines.^27^ However, the study also revealed that in an endeavour to offer improved access to care, some healthcare facilities are open 24 h a day.^27^ This does not happen everywhere, however, and some facilities continue to render services for 12 h a day only – a situation that should be changed.^28^ Patient waiting time (PWT) surveys are necessary to develop plans to respond better to patient concerns. Section 27 of the South African Constitution^29^ provides that everyone has the right to access healthcare services, including sexual and reproductive health and chronic care services. The study revealed that a number of patients who were stable were not offered decanting services and so continued to experience long waiting hours unnecessarily. A report by Ritshidze project^30^ revealed long waiting hours in most health facilities in South Africa and recommends that the department of health should ensure adequate staffing levels, improve employee attitude, extend opening hours and improve the state of filing systems.

Clinics, community health centres and district hospital outpatient departments are described as being ‘pro-poor’, meaning that these facilities are used more frequently by lower than by higher socio-economic groups.^31^ Only 17 South Africans out of every 100 have medical insurance, which allows them to access private healthcare services. According to the most recent General Household Survey conducted in 2018 and 2021, revealed that up to 45 million South Africans, or 82 out of every 100 people, are not covered by medical aid, making them heavily reliant on the country’s PHC system,^27^ and at the time of writing, national health insurance had not yet been implemented; the study revealed a high number of patients without medical aid or health insurance because they were unemployed and thus denied access to better services and expert care.

### Mental health problems

The findings of the study revealed that PWCDs experienced mental health problems such as fear, stress, anxiety and depression, and that psychosocial support was lacking, as family visits were restricted and community-based support systems such as adherence clubs were disbanded as a result of the COVID-19 pandemic. This was supported by the WHO^32^ survey in 2020, which noted that community-based psychotherapy and counselling interventions were severely disrupted; however, countries still need to adapt to ensure the continuity of mental health services. This should relieve the pressure on the already overburdened healthcare system, including external pick-up points for patients who are stable in response to treatment. Patients with chronic diseases cannot rely exclusively on their family and buddy for support but should meet other patients to share experiences, learn from one another and support one another.^31^

A study conducted by Whittington et al.^33^ indicated that key and vulnerable groups such as members of the lesbian, gay, bisexual, transgender, queer, intersex, and asexual (LGBTQI+) community and women were also affected by the pandemic. The study revealed serious mental health problems among PWCDs as a result of the COVID-19 pandemic, with patients living in fear of contracting COVID-19 infections and developing complications related to comorbidity. Patients also suffered from stress and depression; however, some developed greater resilience and coping abilities during these difficult times. This is supported by Gobeil-Lavoie et al.,^34^ who express the view that PWCDs run the risk of developing depression, experiencing psychological distress, having low self-efficacy and receiving conflicting information from medical professionals. Furthermore, Addis et al.^35^ indicated the extreme psychological effects of COVID-19 in a 22.8% (confidence interval [CI]: 18.6–27.1) of individuals with chronic diseases. A correlation was drawn between age, gender, the severity of the patient’s chronic illness, respiratory problems and a lack of social support and negative psychological effects.^35^

### Socio-economic impact

High levels of unemployment were reported among PWCDs and their relatives. The COVID-19 pandemic influenced the lives of so many, with the effects on PWCDs being more severe, as their health limits their capacity to compete in the labour market. Patients lost their employment and opportunities to sell vegetables and fruit; they, therefore, struggled to buy food, medication and supplies for self-care. This was borne out by a study conducted in India, China, Korea and Hong Kong by Dubey et al.^36^ and Alex et al.,^37^ which indicated that between 19% and 59% of the participants reported their financial situation as having worsened during the COVID-19 pandemic. Of the interviewees, 8% – 24% reported increased difficulty in obtaining access to care, with 5.6% – 14.6% of participants stating that their diabetic symptoms were worsening, with economic hardship affecting these patients in terms of maintaining a balanced diet and buying other required medical supplies.^38^ Another study revealed that unemployment also contributes to mental health problems such as stress and depression among PWCDs and is related to self-care needs. This was supported by a study conducted by Yildiz et al.,^39^ indicating that people with chronic diseases in all age groups had lower rates of paid employment and that among those with common mental illnesses, the effects of chronic disease on population-level unemployment were greatest (PAF 0.20).

### Difficulties in accessing healthcare services

The study revealed that PWCDs encountered serious barriers to accessing healthcare services and that this group felt that their needs, rights and expectations in respect of healthcare were not met. According to the WHO,^40^ the provision of high-quality healthcare services must be a global priority for universal health coverage. The burden of disease continues to be a public health concern and affects the healthcare system and the economy of the country. This is borne out by the world bank group OECD,^41^ which makes the observation that because of the high patient load, waiting times, poor communication with experts with regard to coordinating care and resource limitations while managing complex patients, it was difficult to provide safe and high-quality care with short waiting times. Although healthcare professionals were able to give patients self-management and lifestyle guidance, patients’ actions are affected by a range of variables, such as their work demands, beliefs and environment. Baker and Walker^42^ indicated that extended opening hours at primary healthcare facilities are beneficial for patients and not a distraction for health professionals as they usually perceive it as such. As indicated by Sigh et al.,^43^ 83% of those affected by the COVID-19 lockdowns in India reported difficulties in accessing healthcare; 17% experienced difficulty in accessing medications; 59% reported a loss of income, 38% experienced job losses and 28% reduced their intake of fruits and vegetables.

In another study conducted by Westgard et al.,^44^ participants reported a moderately high rate of healthcare use. Moreover, the patients identified several individual and contextual-level factors that may have influenced their experiences and the health-seeking behaviours of their communities, including embarrassment, fear and trust, a lack of healthcare personnel and a lacklustre attitude towards their jobs, a limited supply of essential medications and supplies in the medical facility and a lack of basic medicines and materials.^45^ Dubey et al.^36^ revealed chronic care management and implementation of self-care to be key during difficult times, such as the COVID-19 pandemic. Self-care management of chronic illness is the most cost-effective way to manage chronic disease and has several advantages, including empowering patients by improving self-agility to promote healthy lifestyles and reducing the use of healthcare services, thus lowering healthcare costs.^36^ Regardless of the patient’s location or the care environment, novel patient education strategies must be investigated. Patients and their caregivers should be well informed about treatment options, choices and related risks and benefits before being included as decision-making partners. Nurses should be educators and change-makers.^45^

## Conclusion

The study reported on in this article was conducted to explore the experiences of PWCDs during the COVID-19 pandemic and revealed distressing experiences that affected self-care management and the implementation of the chronic care model. Limited access to quality management of the chronic disease will worsen the burden of diseases globally and continue to affect the healthcare system, which buckled under the pressure of the COVID-19 pandemic. The complications that might result from poor chronic care have the potential to affect a patient’s prognosis and life expectancy and the cost of healthcare services. The needs and expectations of PWCDs cannot be underestimated and are key considerations when dealing with difficulties in accessing healthcare services and the hardships occasioned by the COVID-19 pandemic. The findings of the study could influence policy governing the management of chronic diseases during future epidemics. They may also provide healthcare providers with evidence-based practice and guidance on how to review and improve the chronic care model based on the experiences of PWCDs, with the goal of improving patient health outcomes and satisfaction with the healthcare services provided. If the chronic care model is properly implemented, it will eventually reduce the disease burden and lower the costs associated with managing chronic disease complications.

Based on the study’s findings, policymakers should consider giving PWCDs priority by teaching them evidence-based self-care approaches, strengthening psychosocial support to deal with anxiety and fear of co-morbidity-related complications, maximise the implementation of patient-centred interventions, the appointment system, decanting stable patients to enhance chronic care management model. Non-communicable diseases should be considered as a daily public health emergency.

## References

[1] World Health Organization. Integrated chronic disease prevention and control. Geneva: World Health Organization; 2021.

[2] World Health Organization. Preventing chronic disease is a vital investment. Geneva: World Health Organization; 2020.

[3] Joint United Nations programme on HIV/AIDS: (UNIAIDS) State of epidemic data. Geneva: UNIAIDS; 2020.

[4] WHO. Noncommunicable diseases fact sheet [homepage on the Internet]. 2022 [cited 2022 Dec 14]. Available from: https://www.who.int/news-room/fact-sheets/detail/noncommunicable-diseases

[5] Organisation for Economic Co-operation and Development (OECD). The territorial impact of COVID-19: Managing the crisis across levels of government [homepage on the Internet]. 2022 [cited 2022 Dec 14]. Available from: https://www.oecd.org/coronavirus/policy-responses/the-territorial-impact-of-COVID-19-managing-the-crisis-across-levels-of-government-d3e314e1/

[6] Mbunge E. Effects of COVID-19 in South African health system and society: An explanatory study. DiabetesMetab Syndr. 2020;14(6):1809–1814. 10.1016/j.dsx.2020.09.016 PMC7485444 32956925

[7] World Health Organization. Non-communicable diseases in emergencies. Geneva: WHO; 2016.

[8] World Health Organization. HIV: From a devastating epidemic to a manageable chronic disease [homepage on the Internet]. Geneva: World Health Organization; 2017 [cited 2022 Jul 26]. Available from: https://reliefweb.int/report/world/hiv-devastating-epidemic-manageable-chronic-disease

[9] Organisation for Economic Co-operation and Development (OECD). COVID-19 in Africa: Regional socio-economic implications and policy priorities [homepage on the Internet]. [cited 2022 Dec 14]. Available from: https://www.oecd.org/coronavirus/policy-responses/covid-19-and-africa-socio-economic-implications-and-policy-responses-96e1b282/

[10] Joint United Nations programme on HIV/AIDS: (UNAIDS) Global HIV/AIDS response progress report. Geneva; 2011.

[11] National Institute for Communicable Diseases. The first case of COVID-19 coronavirus was reported in SA [press release]. Pretoria; 2022 [cited 2022 Jul 25]. Available from: https://www.nicd.ac.za/first-case-of-covid-19-coronavirus-reported-in-sa/

[12] OECD, WHO, World Bank. EU integrated approach to non-communicable diseases [homepage on the Internet]. 2020 [cited 2022 July 06]. Available from: https://ec.europa.eu/health/non_communicable_diseases/overview_en

[13] South Africa. Department of Health. Message by President Cyril Ramaphosa on COVID-19 pandemic [Press release]. Pretoria; 2020 [cited 2022 Dec 14]. Available from: https://sacoronavirus.co.za/2020/04/09/message-by-president-cyril-ramaphosa-on-covid-19-pandemic-thursday-9-april-2020/

[14] LoBiondo-Wood G, Haber J. Nursing research: Methods and critical appraisal for evidence-based practice. 7th ed. New York, NY: Mosby Elsevier; 2010.

[15] Brink H, Van der Walt C, Van Rensburg G. Fundamentals of research methodology for healthcare professionals. 4th ed. Cape Town: Juta; 2018.

[16] Creswell JW, Creswell JD. Research design: Qualitative, quantitative, and mixed methods approaches. 5th ed. Los Angeles, CA: Sage; 2018.

[17] Gray JR, Grove SK, Sutherland S. The practice of nursing research. 8th ed. St Louis Missouri: Elsevier; 2018.

[18] Polit DF, Beck CT. 2017. Nursing research. Generating and assessing evidence for nursing practice. 9th ed. Philadelphia: W & W Lippincott.

[19] Starkman E. Critical, stable, or fair: Defining patient conditions, American hospital association. 2022 [cited 2022 Dec 14]. Available from: https://www.webmd.com/a-to-z-guides/defining-patient-conditions

[20] Andersen RM, Newman JF. Revisiting the behavioural model of access to medical care: Does it matter? J Health Soc Behav. 1995;36(1):1–10. 7738325

[21] De Vos AS, Strydom H, Fouche CB, Delport CSL. Research at the grassroots. Pretoria: Van Schaik; 2017.

[22] Syed MA, Alnuaimi AS, Zainel AJ, Qotba HA. Prevalence of non-communicable diseases by age, gender, and nationality in publicly funded primary care settings in Qatar. BMJ Nutr Prev Health. 2019;2(1):20–29. 10.1136/bmjnph-2018-000014 PMC7678476 33235953

[23] Belaunzaran-Zamudio PF, Caro-Vega Y, Giganti MJ, et al. Frequency of non-communicable diseases in people 50 years of age and older receiving HIV care in Latin America. PLoS One. 2020;15(6):e0233965. 10.1371/journal.pone.0233965 32555607 PMC7299309

[24] Roy CM, Bukuluki P, Casey SE, et al. Impact of COVID-19 on gender-based violence prevention and response services in Kenya, Uganda, Nigeria, and South Africa: A cross-sectional survey. Front Glob Women’s Health. 2022;2:780771. 10.3389/fgwh.2021.780771 35156086 PMC8829509

[25] Liang Y, Zhao L. Intelligent hospital appointment system based on health data. Procedia Computer Science. 2019;159:1880–1889.

[26] South Africa. Department: Statistics South Africa. General household survey. Population suffering from a chronic health condition. Pretoria; 2022 [cited 2022 Sep 24]. Available from: https://www.statssa.gov.za/?p=15482

[27] South Africa. Department: Statistics South Africa. Population suffering from a chronic health condition. Medical aid scheme. Pretoria: Government printer; 2022.

[28] South Africa. Department of Health. National guideline for management of patient waiting times at health facilities. Pretoria: Government printer; 2019.

[29] Constitutional assembly. South Africa, Constitution of the Republic of South Africa Act no 108 of 1996. Pretoria: Government printer; 1996.

[30] Ritshidze Report. State of health in Gauteng, saving our lives, South Africa [homepage on the Internet]. ritshidze.org.za. 2022 [cited 2022 Sep 24]. Available from: https://ritshidze.org.za/wp-content/uploads/2020/11/Ritshidze-State-of-Gauteng-Report-Dec-2020.pdf

[31] Eaton J, Rahman A, Gater R, Saxena S, Hammerich A, Saeed K. From adversity to resilience in the COVID-19 era: Strengthening mental health systems in the Eastern Mediterranean Region. East Mediterr Health J. 2020;26(10):1148–1150. 10.26719/2020.26.10.1148 33103740

[32] WHO. COVID-19 disrupting mental health services in most countries, WHO survey [homepage on the Internet]. 2020 [cited 2022 Dec 14]. Available from: https://www.who.int/news/item/05-10-2020-covid-19-disrupting-mental-health-services-in-most-countries-who-survey

[33] Whittington C, Hadfield K, Calderón C. The lives and livelihoods of many in the LGBTQ + community are at risk amidst the COVID-19 crisis. Human Rights Campaign: Germany; 2020, p. 1–7.

[34] Gobeil-Lavoie AP, Chouinard MC, Danish A, et al. Characteristics of self-management among patients with complex health needs: A thematic analysis review. BMJ Open. 2019;9:e028344. 10.1136/bmjopen-2018-028344 PMC6538095 31129599

[35] Addis SG, Nega AD, Miretu DG. The psychological impact of the COVID-19 pandemic on chronic disease patients in Dessie town government and private hospitals, Northeast Ethiopia. Diabetes Metab Syndr. 2021;15(1):129–135. 10.1016/j.dsx.2020.12.019 33338951 PMC7728425

[36] Dubey S, Biswas P, Ghosh R, et al. Psychosocial impact of COVID-19. Diabetes Metab Syndr. 2020;14(5):779–788. 10.1016/j.dsx.2020.05.035 32526627 PMC7255207

[37] Alex J, Ramjan L, Salamonson Y, Ferguson C. Nurses as key advocates of self-care approaches to chronic disease management. Contemp Nurse. 2020;56(2):101–104. 10.1080/10376178.2020.1771191 32552496

[38] Singh K, Kondal D, Mohan S, et al. Health, psychosocial, and economic impacts of the COVID-19 pandemic on people with chronic conditions in India: A mixed methods study. BMC Public Health. 2021;21(1):685. 10.1186/s12889-021-10708-w 33832478 PMC8027966

[39] Yildiz B, Burdorf A, Schuring M. The influence of chronic diseases and multimorbidity on entering paid employment among unemployed persons – A longitudinal register-based study. Scand J Work Environ Health. 2021;47(3):208–216. 10.5271/sjweh.3942 33350454 PMC8126442

[40] WHO. Universal health coverage (UHC) [homepage on the Internet]. 2021 [cited 2022 Aug 23]. Available from: https://www.who.int/news-room/fact-sheets/detail/universal-health-coverage-(uhc)

[41] World Bank Group, OECD. Delivering quality health services [homepage on the Internet]. World Health Organization: Switzerland; 2018, p. 1–100. Available from: http://apps.who.int/bookorders

[42] Baker R, Walker N. Extended opening hours in primary care: Helpful for patients and/or a distraction for health professionals? BMJ Qual Saf. 2017;26(5):347–349. 10.1136/bmjqs-2016-005415 27435190

[43] Singh et al. Impact of the COVID-19 pandemic on chronic disease care in India, China, Hong Kong, Korea, and Vietnam. Asia Pacific J Publ Health. 2022;34(4): 392–400. 10.1177/10105395211073052 PMC9133173 35067078

[44] Westgard CM, Rogers A, Bello G, Rivadeneyra N. Health service utilization, perspectives, and health-seeking behavior for maternal and child health services in the Amazon of Peru, a mixed-methods study. Int J Equity Health. 2019;18(1):155. 10.1186/s12939-019-1056-5 31615516 PMC6794768

[45] Foo KM, Sundram M, Legido-Quigley H. Facilitators, and barriers of managing patients with multiple chronic conditions in the community: A qualitative study. BMC Public Health. 2020;20(1):273. 10.1186/s12889-020-8375-8 32106838 PMC7045577

